# MicroRNA-19a regulates lipopolysaccharide-induced endothelial cell apoptosis through modulation of apoptosis signal-regulating kinase 1 expression

**DOI:** 10.1186/s12867-015-0034-8

**Published:** 2015-05-16

**Authors:** Wei-Long Jiang, Yu-Feng Zhang, Qing-Qing Xia, Jian Zhu, Xin Yu, Tao Fan, Feng Wang

**Affiliations:** Department of Respiration, Jiangyin Hospital of Traditional Chinese Medicine Affiliated to Nanjing University of Chinese Medicine, Jiangyin City, Jiangsu Province 214400 China; Department of Neurology, Jiangyin Hospital of Traditional Chinese Medicine Affiliated to Nanjing University of Chinese Medicine, Jiangyin City, Jiangsu Province 214400 China; Department of Internal Medicine, Jiangyin Hospital of Traditional Chinese Medicine Affiliated to Nanjing University of Chinese Medicine, Jiangyin City, Jiangsu Province 214400 China; Department of Neurology, Shanghai First People’s Hospital, Shanghai Jiaotong University School of Medicine, Shanghai, 200080 China

**Keywords:** miR-19a, ASK1, Apoptosis, Endothelial cells

## Abstract

**Background:**

MicroRNAs, small non-encoding RNAs that post-transcriptionally modulate expression of their target genes, have been implicated as critical regulatory molecules in endothelial cells.

**Results:**

In the present study, we found that overexpression of miR-19a protects endothelial cells from lipopolysaccharide (LPS)-induced apoptosis through the apoptosis signal-regulating kinase 1 (ASK1)/p38 pathway. Quantitative real-time PCR demonstrated that the expression of miR-19a in endothelial cell was markedly down-regulated by LPS stimulation. Furthermore, LPS-induced apoptosis was significantly inhibited by over-expression of miR-19a. Finally, both a luciferase reporter assay and western blot analysis showed that ASK1 is a direct target of miR-19a.

**Conclusions:**

MiR-19a regulates ASK1 expression by targeting specific binding sites in the 3’ untranslated region of ASK1 mRNA. Overexpression of miR-19a is an effective method to protect against LPS-induced apoptosis of endothelial cells.

**Electronic supplementary material:**

The online version of this article (doi:10.1186/s12867-015-0034-8) contains supplementary material, which is available to authorized users.

## Background

MicroRNAs (miRNAs) are endogenous, small non-coding RNA molecules consisting of about 22 nucleotides, which function in RNA silencing and post-transcriptional regulation of gene expression [1-4]. Many miRNAs are evolutionarily conserved and believed to play a role in controlling various biological process including developmental patterning, cell differentiation, and cell proliferation [5-7]. MiR-19a belongs to the MiR-17-92 cluster that encodes six single mature miRNAs (miR-17, miR-19a/b, miR-20, miR-92, and miR18) [8-10]. It is up-regulated in a variety of cancers including gliomas, medulloblastoma, gastric cancer, and thyroid cancer, and enhances proliferation, inhibits apoptosis, and induces tumor angiogenesis, indicating that miR-19a is an oncogene [11-17]. MiR-19a is also involved into controlling endothelial cell functions and neovascularization [18,19]. It has been reported that miR-19a expression increases during induction of endothelial cell differentiation in embryonic stem cells [20].

Recently, Philippe et al. reported that lipopolysaccharide (LPS) down-regulates the expression of miR-19a and miR-19b, which is associated with toll-like receptor 2 up-regulation [21]. It is well known that LPS induces apoptosis in various types of endothelial cells including human umbilical vein endothelial cells (HUVECs) and lung-derived normal human microvascular endothelial cells [22-24]. Previous studies have also reported that LPS release into circulation induces endothelial cell apoptosis in vivo and thus causes microvascular injury in numerous tissues [25-27]. LPS induces the activity of apoptosis signal-regulating kinase 1 (ASK1) and activates the downstream mitogen-activated protein kinase (MAPK) pathways, leading to induction of JNK/p38 activity and resulting in apoptosis [28]. ASK1-deficient mice have been shown to be resistant to LPS-induced sepsis shock [29]. LPS-induced p38 activation and production of inflammatory cytokines are reduced in splenocytes and dendritic cells derived from ASK1-deficient mice [29]. As a member of the MiR-17-92 cluster, miR-20 has been also reported to target ASK1 [9]. Therefore, it might be interesting to determine whether miR-19a and miR-20 share a common mechanism in LPS-induced apoptosis.

In the present study, we identified miR-19a, whose expression was markedly down-regulated in LPS-stimulated HUVECs, as a novel modulator of ASK1 expression and LPS-induced endothelial cell apoptosis.

## Methods

### Cells and reagents

HUVECs and EAhy926 cells were purchased from the American Type Culture Collection (Manassas, VA, USA). A miRNA-19a inhibitor (Product Number: HSTUD0343) and control inhibitor (Product Number: NCSTUD001) were purchased from Sigma-Aldrich. The miRNA inhibitors were designed using the mature miRNA sequence information from miRBase and are 2’-O-methylated RNA duplexes with a miRNA-binding site on each strand.

### Western blotting

To assess ASK1 expression, proteins from HUVECs were collected and analyzed by western blotting. Briefly, a protein sample (20 μg) was fractionated by SDS-polyacrylamide gel electrophoresis and then transferred to a polyvinylidene difluoride membrane (Immobilon-P; Millipore). The membrane was blocked with phosphate-buffered saline containing 0.3% Tween 20 and 5% dry milk, and then incubated with a primary antibody overnight at 4°C. The immune complexes were detected by chemiluminescence methods (ECL; Amersham International). Anti-ASK1 and anti-phospho-ASK1(Thr845) antibodies were purchased from abcam. Anti-p38, anti-phospho-p38, anti-cleaved caspase-3, and anti-glyceraldehyde-3-phosphate dehydrogenase (GAPDH) antibodies were purchase from Cell Signaling Technology. All antibodies were diluted at 1:1000. GAPDH was used as a loading control.

### Generation of a miR-19a adenovirus

The miR-19a adenovirus used in this study contained the human miR-19a gene (NR_029489.1). The adenovirus was generated using the AdMax (Microbix) system according to the manufacturer’s recommendations. Briefly, the pacAd5 9.2-100 Ad backbone vector was cotransfected with the pacAd5 K-NpA shuttle vector containing the miR-19a sequence into Ad293 cells using FuGene 6 Transfection Reagent (Roche, Indianapolis, IN). The viruses were propagated in Ad293 cells and purified using Cscl_2_ banding followed by dialysis against 10 mmol/L Tris-buffered saline with 10% glycerol. Titering was performed with HEK293 cells using an Adeno-X Rapid Titer kit (BD Biosciences Clontech, Palo Alto, CA) according to the manufacturer’s instructions. An adenovirus bearing LacZ (Ad-LacZ) was obtained from Clontech.

### Quantitative real-time PCR

Total RNAs were extracted from HUVECs using a miRNeasy Mini Kit or RNeasy kit (QIAGEN). Quantitative real-time PCR (qRT-PCR) was performed with cDNA generated from 20 ng total RNA using a miRCURY LNATM Universal cDNA Synthesis kit and SYBR® Green Master Mix Kit (Exqion). MiR-19a primers were 5’-CCTCTG-TTAGTTTTGCATAGTTGC-3’ and 5’-CAGGCCACCATCAGTTTTG-3’; miR-20a primers were 5’-ACACTCCAGCTGGGTAAAGTGCTTATAGTGC-3’ and 5’-CTCAACTGGTGTCGTGGAGTCGGCAATTCAGTTGAGCTACCTGC-3’ (stem-loop reverse primer). qRT-PCR analysis of ASK1 expression was performed with cDNA generated from 250 ng total RNA using HotStart-IT® SYBR® Green qPCR Master Mix with a UDG (2×) Tested User FriendlyTM kit (USB Corporation). ASK1 primers were 5’-AGACATCTGGTCTCTGGGCTGTAC-3’ and 5’-AACATTCCCACCTTGAACATAGC-3’. The relative expression level was calculated by the 2-ΔΔCt method with the CT values normalized to 18S rRNA as the internal control for ASK1 and U6 snRNA as the internal control for miRNAs.

### Luciferase reporter assay

Based on the human ASK1 mRNA sequence, firefly luciferase cDNA fused with the human ASK1 mRNA 3’ untranslated region (UTR) containing the two seed sequences for miR-19a was amplified from the genomic DNA of HUVECs (two primers containing XbaI sites were used. Forward: 5’-TGTAGAGTTGAGAGTCTCTTTAATT-3’; Reverse: 5’-TGTAGACTGTTGCTCAATCTAATCTTC-3’). The ASK 3’UTR was cloned into pGL3-promoter luciferase reporter vector(Promega, Madison, USA) between luciferase coding sequence and SV40-poly(A) sequence using the XbaI site.Two miR-19a sites located in the ASK1 3’UTR (site-1: UUGCAC, starting at nt 304, and site-2: UUGCAC, starting at nt 620) were mutated to Luc-ASK1 3’UTR MU (UUCGTG) using a QuickChange II Site-Directed Mutagenesis Kit (Agilent Technologies, Santa Clara, CA). Specific primers were used for mutagenesis of miR-19a target site-1 and −2 in the human ASK1 3’UTR (site-1 forward: 5’-CAGCAGCTATTCGTGTTCAGCC-3’, site-1 reverse: 5’-GGCTGAACACGAATAGCTGCTG-3’; site-2 forward: 5’-ACTGTACCAGTTCGTGATGCTTGA-3’, site-2 reverse: 5’-CAAGCATCACGAACTGGTACAGT-3’). For the reporter gene assay, EAhy926 cells were cultured in 12-well plates, and transduced with Ad-LacZ and Ad-miR-19a for 24 h. Then, the cells were transfected with 300 ng firefly luciferase reporter plasmid (pGL3-Luc-ASK1 3’UTR or pGL3-Luc-ASK1 3’UTR MU) and 20 ng Renilla luciferase reporter plasmid pRL-RSV (Promega) using Lipofectamine 2000 transfection reagent (Invitrogen). Luciferase assays were performed at 48 h after transfection using the Dual Luciferase Reporter Assay system (Promega Biotech Co., Ltd). Firefly luciferase activities were normalized to Renilla luciferase activities.

### Sandwich enzyme-linked immunosorbent assay for histone-associated DNA fragments

Endothelial cell death was assessed by an enzyme-linked immunosorbent assay using a Death Detection Kit from Boehringer Mannheim (Indianapolis, IN). HUVECs were seeded at 2 × 10^4^ cells per well in a 96-well plate and grown to 90% confluence. The cells were then treated with LPS (100 ng/ml) for the indicated times. The cells were harvested in lysis buffer, and the cytoplasmic and nuclear protein fractions were separated by centrifugation at 200 × *g*. The supernatant (cytoplasmic fraction) was used to measure histone-associated DNA fragments.

### Statistical analyses

Data are expressed as means ± standard error. The statistical significance of differences was assessed by Student’s t-tests or analysis of variance as appropriate. A value of *P* < 0.05 was considered statistically significant.

## Results

### LPS down-regulates miR-19a expression in endothelial cells

**Figure 1:**
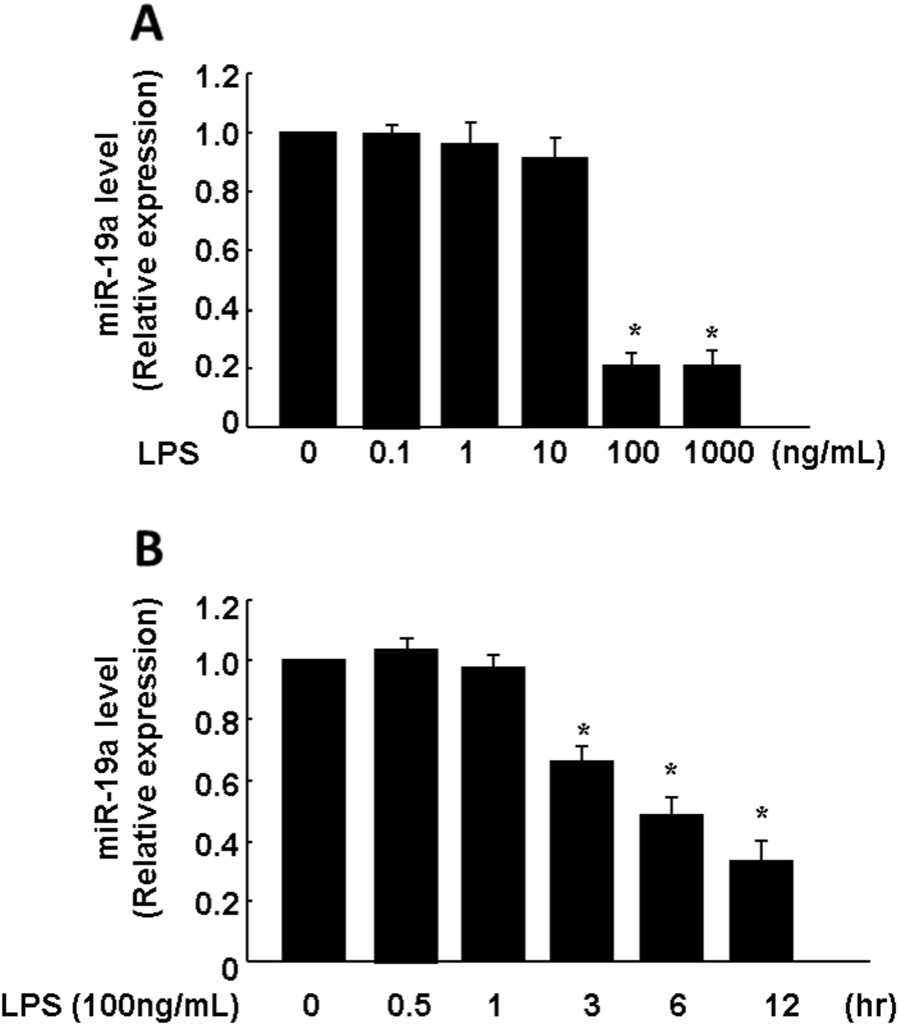
LPS down-regulates expression of miR-19a in endothelial cells. **(A)**, HUVECs were cultured in 6-well plate, after 80% confluence, cells were treated with different concentration of LPS for 12 h. **(B)**, HUVECs were treated with 100 ng/mL LPS for different times point as indicated. Quantitative real-time PCR showed that miR-19a expression was down-regulated, compared with that in the respective control group. Significance is indicated as *P < 0.05.

The human ASK1 3’-UTR was analyzed using a website tool (http://www.microrna.org/microrna/getGeneForm.do). As a result, we found ASK1 target miRNAs (Additional file 1: Figure S1). To investigate whether miR-19a expression is altered during LPS-induced endothelial cell apoptosis, HUVECs were treated with various doses of LPS for the indicated times. The expression level of miR-19a was detected by qRT-PCR. As shown in Figure 1, miR-19a expression was not affected by an LPS concentration of less than 10 ng/ml. However, miR-19a expression was significantly decreased by 100 ng/ml LPS (Figure 1A). In addition, we found a decrease in miR-19a expression by about 80% after treatment with 100 ng/ml LPS for 12 h (Figure 1B). Taken together, these data show that LPS down-regulates miR-19a expression in a dose and time-dependent manner.

### Expression of ASK1 is up-regulated during LPS-induced endothelial cell apoptosis

**Figure 2:**
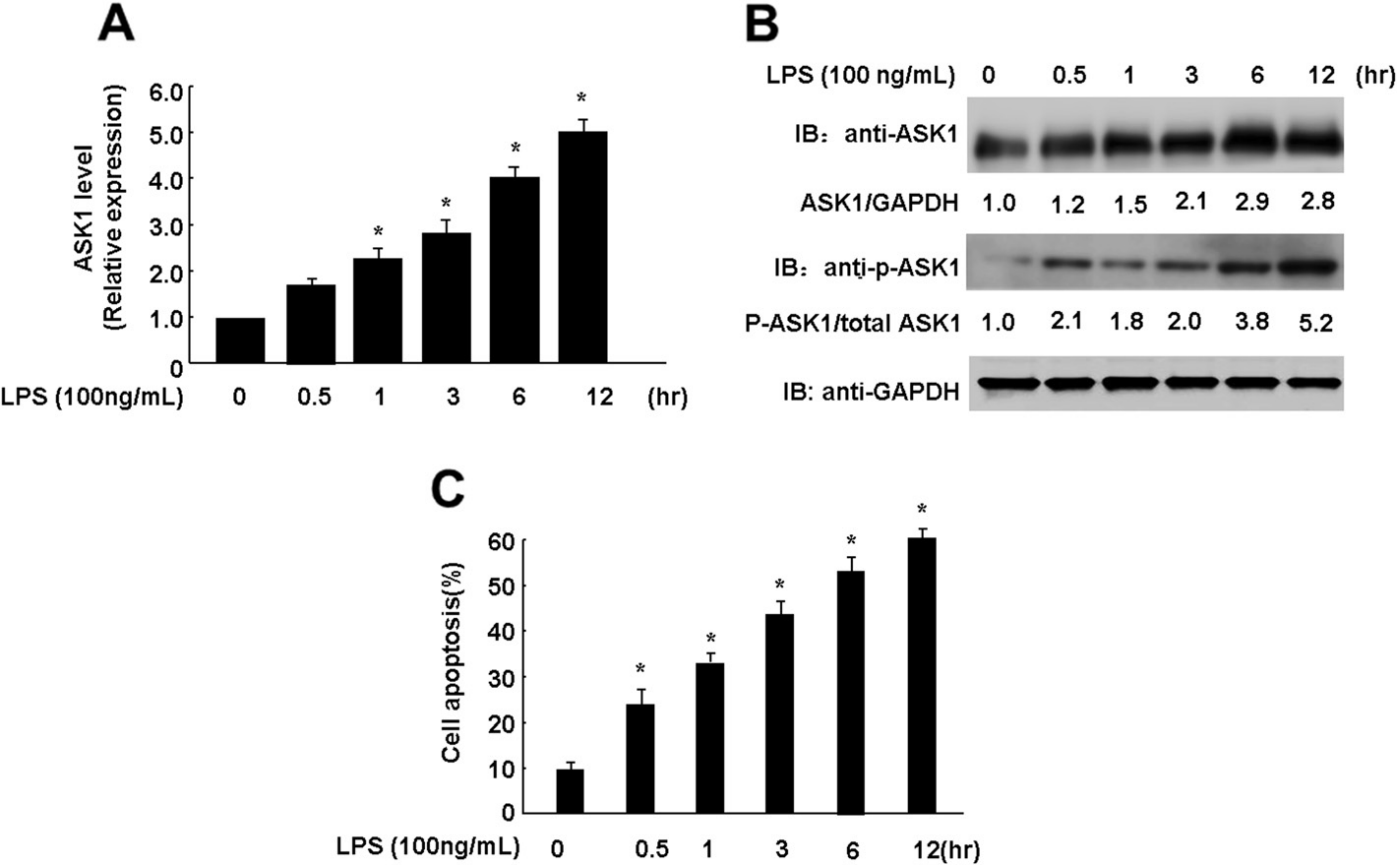
Expression of ASK1 was up-regulated during LPS-induced endothelial cell apoptosis. **(A)**, HUVECs were cultured in 6-well plate, after 80% confluence, cells were treated with 100 ng/mL LPS for different times point as indicated. Quantitative real time PCR revealed that the expression levels of ASK1 were increased after LPS stimuli. **(B)**, Cell lysates obtained from HUVECs were subjected to western blot. ASK1, phosphorylation of ASK1 at Thr845, and GAPDH were determined by indicated antibodies. **(C)**, the quantitative analysis of HUVECs apoptosis was determined by ELISA assay. Significance is indicated as *P <0.05.

To examine the possible link between miR-19a and ASK1, we also measured the expression of ASK1 at both mRNA and protein levels. HUVECs were stimulated for 12 h with LPS, and then qRT-PCR was performed using RNA isolated from control and treated cells. We observed a 2–5-fold increase of Ask1 transcripts in response to LPS (Figure 2A). To determine whether the increase in Ask1 mRNA expression correlated with enhanced ASK1 protein expression, we performed western blotting and quantified ASK1 expression by densitometry. As shown in Figure 2B, treatment of HUVECs with LPS led to increased expression of ASK1 by up to 2.8-fold. ASK1 can be phosphorylated at several sites, and these phosphorylation sites regulate ASK1 activity in both positive and negative manners. Phosphorylation of ASK1 at Ser83 inhibits ASK1-induced apoptosis. On the other hand, phosphorylation of ASK1 at Thr845 promotes ASK1-induced apoptosis [30,31]. The activity of ASK1 induced by LPS was indicated by detecting phosphorylation of ASK1 at Thr845. As shown in Figure 2B, LPS significantly increased the phosphorylation of ASK1 at Thr845 by up to 5.2-fold. Finally, we detected LPS-induced HUVEC apoptosis by a TUNEL assay. As expected, LPS treatment resulted in an increase of HUVEC apoptosis, which is consistent with other studies [32,33].

### Over-expression of miR-19a attenuates LPS-induced apoptosis in endothelial cells

**Figure 3:**
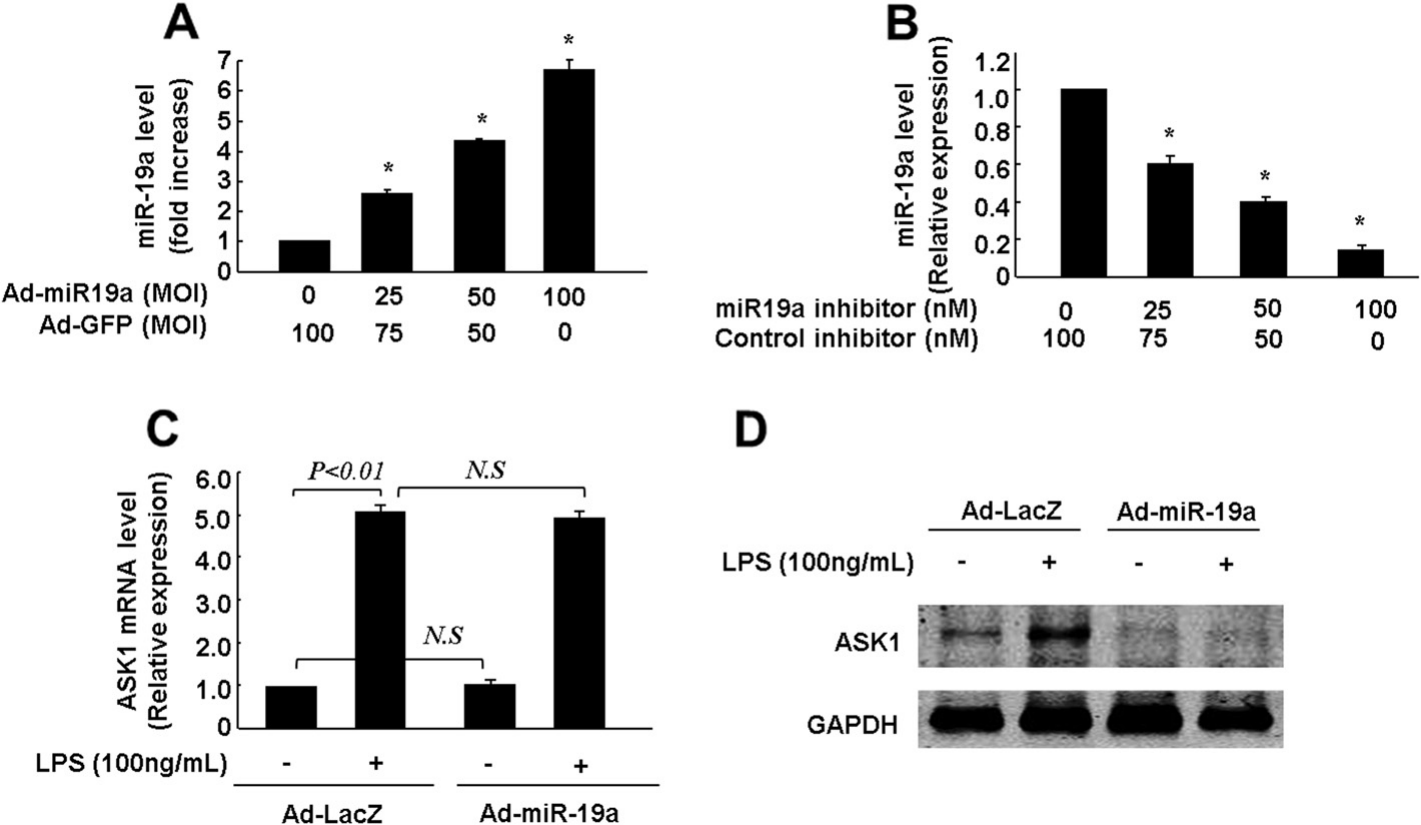
Expression of miR-19a in HUVECs either transduced with Ad-miR19a and transfected with miR-19a inhibitor. **(A)** and **(B)**, Quantitative real time PCR revealed that the expression levels of miR-19a were increased after Ad-miR-19a transduction **(A)**, but suppressed by miR-19a inhibitor in a dose-dependent manner **(B)**. Significance is indicated as *P < 0.05. **(C)** and **(D)**, MiR-19a regulates ASK1 expression. HUVECs were transduced with Ad-miR-19a or Ad-LacZ (50MOI) for 24 h, and then treated with 100 ng/mL LPS for another 24 h. The expression of ASK1 was determined by quantitative real time PCR **(C)** and western blot **(D)**.

We next tested whether overexpression of miR-19a affected ASK1 expression levels and apoptosis in endothelial cells. To this end, we generated Ad-miR19a. First, qRT-PCR was performed to evaluate miR-19a expression levels in HUVECs transduced with either Ad-miR-19a or transfected with the miR-19a inhibitor. Transduction of HUVECs with Ad-miR-19a led to a marked increase of miR-19a levels in a dose-dependent manner (Figure 3A). However, transfection of the miR-19a inhibitor significantly suppressed miR-19a expression (Figure 3B), but had no effect on miR-20a expression (Additional file 1: Figure S2). Second, we evaluated the expression of ASK1 in LPS-treated HUVECs. We found that LPS treatment significantly induced ASK1 expression at the mRNA level, but the up-regulation of Ask1 mRNA was not affected by overexpression of miR-19a (Figure 3C). Interestingly, overexpression of miR-19a by the adenovirus resulted in marked down-regulation of LPS-induced ASK1 expression at the protein level (Figure 3D). These results suggest that miR-19a regulates the expression of ASK1 at the translational level.

**Figure 4:**
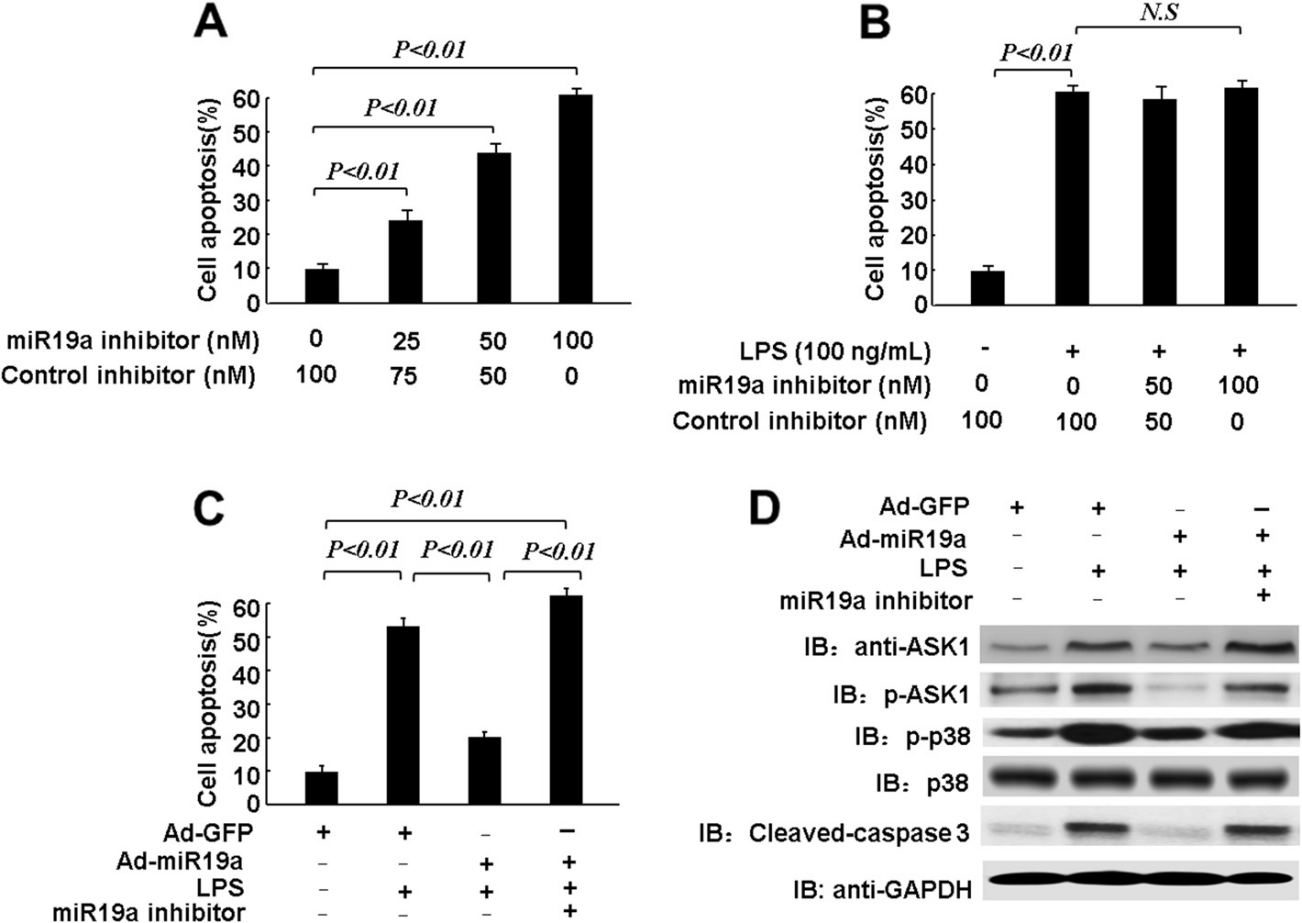
Over-expressing miR-19a attenuated LPS-induced Apoptosis in endothelial cells. **(A)**, MiR-19a inhibitor induces HUVECs apoptosis. HUVECs were transduced with miR19a inhibitor or Control inhibitor for 48 h, then cell apoptosis was determined by ELISA assay. **(B)**, HUVECs were transduced with miR19a inhibitor or Control inhibitor for 24 h, then treated with 100 ng/mL LPS for another 24 h. Cell apoptosis was determined by ELISA assay. **(C)**, HUVECs were transduced with Ad-miR-19a or miR19a inhibitor or Ad-GFP (50MOI) for 24 h, then treated with 100 ng/mL LPS for another 24 h. Cell apoptosis was determined by ELISA assay. **(D)**, Cell lysates obtained from HUVECs were subjected to western blot. ASK1, phosphorylation of ASK1 at Thr845, p38, phosphorylation of p38, cleaved caspase3 and GAPDH were determined by indicated antibodies.

To investigate the effects of miR-19a on HUVEC apoptosis, we used Ad-miR-19a and the miR-19a inhibitor. We found that the miR-19a inhibitor increased HUVEC apoptosis in a dose-dependent manner. However, the miR-19a inhibitor had no effect on LPS-induced HUVEC apoptosis (Figure 4A and B). Over-expression of miR-19a substantially inhibited LPS-induced HUVEC apoptosis, but this effect was reversed by miR-19a inhibitor treatment, further indicating the involvement of miR-19a in LPS-induced endothelial cell apoptosis (Figure 4C). Because ASK1 is directly involved in the LPS-induced apoptotic pathway, we determined whether over-expression of miR-19a regulates the ASK1/p38 apoptotic pathway. We thus performed western blotting to detect the expression levels of key molecules in this apoptotic pathway, including phosphorylated p38 and cleaved caspase-3. The levels of ASK1 and phosphorylation of p38 were significantly increased in cells treated with LPS, but markedly decreased by over-expression of miR-19a (Figure 4D). Indeed, LPS treatment markedly increased the levels of cleaved caspase-3, which were only decreased by miR-19a. However, the decreases in ASK1, phosphorylation of p38, and cleaved caspase-3 due to infection with Ad-miR-19a were significantly reversed by treating the HUVECs with the miR-19a inhibitor (Figure 4D). Taken together, these results support that miR-19a regulates LPS-induced endothelial cell apoptosis through modulation of the ASK1/p38 apoptotic pathway.

### MiR-19a regulates ASK1 expression by targeting its 3’UTR

**Figure 5:**
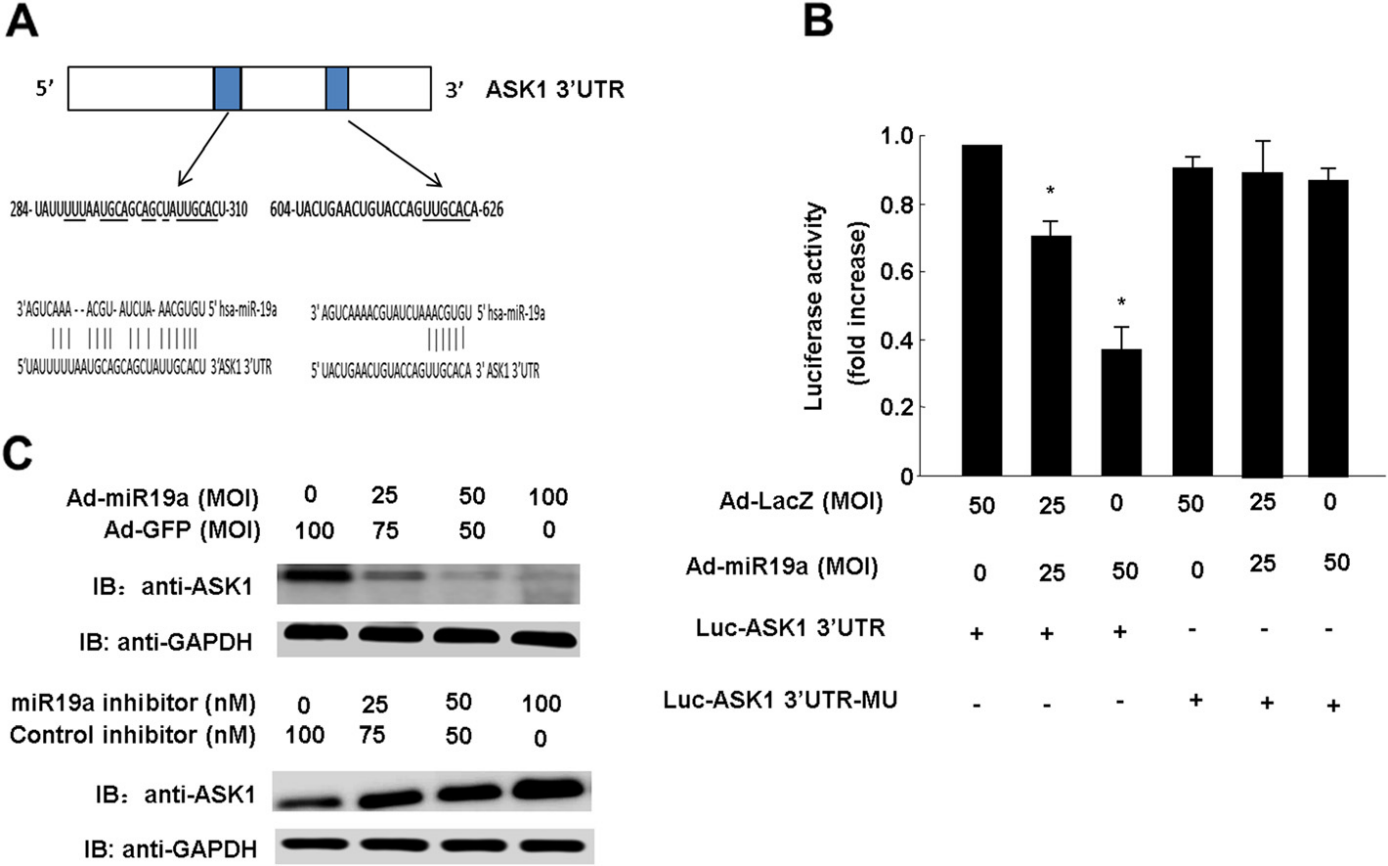
MiR-19a suppressed expression of ASK1 through binding to its 3’UTR. **(A)**, a representative illustration of the putative binding sites for miR-19a in human ASK1 3’UTR. **(B)**, Luciferase assay was performed in EAhy926 cells using pGL3 reporter vector fused with either ASK1 wild-type 3’UTR or ASK1 3’UTR mutant. Over-expression of miR-19a significantly decreased the activity of luciferase gene fused with ASK1 wild-type 3’UTR, but had no effect on the activity of luciferase fused with ASK1 3’UTR mutant. **(C)**, Detection of ASK1 expression by western blot in the whole lysates of HUVECs transduced with different dosages of AdmiR-19a. The result showed that the protein level of ASK1 was suppressed by miR-19a, however, increased by miR-19a inhibitor, in a dose-dependent manner. Significance is indicated as *P <0.05.

To examine whether ASK1 is a direct target of miR-19a, we employed a luciferase reporter assay. Sequence analysis of the human ASK1 3’UTR revealed two putative miR-19a-binding sites located at 287–309 nt and 620–625 nt (Figure 5A). Accordingly, we constructed a reporter plasmid by cloning the ASK1 3’UTR containing the two putative miR-19a-binding sites into the 3’UTR of a pGL3 vector. Two miR-19a-binding sites located in the ASK1 3’UTR (site-1: UUGCAC and site-2: UUGCAC) were mutated to Luc-ASK1 3’UTR MU (UUCGTG) (Additional file 1: Figure S3). As shown in Figure 5B, over-expression of miR-19a markedly down-regulated the activity of the luciferase gene fused with the wild-type ASK1 3’UTR. In contrast, over-expression of miR-19a barely affected the activity of the luciferase gene fused with the mutant ASK1 3’UTR. The protein levels of ASK1 underwent marked dose-dependent down-regulation in cells transduced with Ad-miR-19a at various multiplicities of infection and significant up-regulation in cells treated with the miR-19a inhibitor (Figure 5C). Taken together, these results indicate that ASK1 is a direct target of miR-19a in endothelial cells. MiR-19a regulates ASK1 expression by targeting specific binding sites in the 3’UTR of ASK1.

## Discussion

It is clear that miRNAs contribute to cell development and regulate many biological processes, including cell proliferation and apoptosis, by acting as oncogenes or tumor suppressor genes [5,34]. MiR-19a has been reported to be involved in control of endothelial cell functions and neovascularization [18,19]. LPS has been shown to contribute to damage observed in various types of endothelium [22,25,26]. The molecular pathways of apoptosis are only just being deciphered in endothelial cells. Choi et al. reported that LPS induces apoptosis in microdermal endothelial cells via recruitment of the adaptor Fas-associated death domain [35]. Luyendyk et al. reported that LPS induces the activity of ASK1 and activates downstream MAPK pathways [28]. LPS-induced p38 activation and apoptosis are reduced in splenocytes and dendritic cells from ASK1-deficient mice [29].

MiR-19a belongs to the miR-17-92 cluster [8,9]. Some members of this cluster are regulated by LPS [9,36]. For example, miR-19a and miR-20 are both involved in LPS-induced apoptosis of rheumatoid arthritis fibroblast-like synoviocytes [9,21]. Interestingly, miR-20 has been reported to target ASK1 [9], which prompted us to determine whether miR-19a regulates ASK1 expression. In the present study, we found that LPS down-regulated the expression of miR-19a and miR-20a (Additional file 1: Figure S4), but the decrease of miR-19a expression was more dramatic than that of miR-20a. These data indicate that miR-19a might play a major role in LPS-induced apoptosis. Indeed, ASK1, also known as mitogen-activated protein kinase kinase kinase 5 (MAP3K5), is a member of the MAPK kinase kinase family [37]. Under stress conditions such as oxidative stress and stimulation tumor necrosis factor-α (TNF-α) or LPS, p38 MAPK can be activated by MAP3K5, followed by activation of the downstream target caspase-3 [29,38]. In fact, over-expression of ASK1 has been reported to induce apoptotic cell death [37]. Under stress conditions, ASK1 is auto-phosphorylated at Thr845, resulting in activation of ASK1 and phosphorylation of p38 [39]. Expression of miR-19a was markedly inhibited by 100 ng/ml LPS. Moreover, 100 ng/ml LPS significantly increased the expression of ASK1 in HUVECs as well as activation of the ASK1/p38 pathway, leading to apoptosis. However, we found that over-expression of miR-19a by Ad-miR-19a inhibited LPS-induced HUVEC apoptosis by decreasing the expression of ASK1 and activity of the ASK1/p38 pathway.

Regulation of ASK1 expression occurs at various levels. At the transcriptional level, Ask1 is a target of the E2F family of transcription factors, but there is currently no evidence that E2F-mediated transcriptional control of Ask1 is involved in the injury-induced expression of Ask1 in vivo [40]. ASK1 expression is also regulated at the post-translational level through either c-IAP1-mediated ubiquitination or a SOCS1-dependent degradation process [41,42]. We found that the LPS-induced up-regulation of ASK1 mRNA was not affected by overexpression of miR-19a (Figure 3C). However, overexpression of miR-19a by the adenovirus impaired LPS-induced ASK1 expression at the protein level (Figures 3D and 4D). These data clearly indicate that miR-19a regulates ASK1 expression at the post-transcription level.

Indeed, regulation of miR-19 in apoptosis has been reported by other studies [8,17]. Interestingly, miR-19a target proteins, such as PTEN, p53, TNF-α and SMAD4, have been reported to regulate apoptosis [12,43-45]. In the present study, we found that miR-19a not only down-regulated the activity of ASK1, but also inhibited the expression of ASK1. These results indicate that miR-19a regulates LPS-induced endothelial cell apoptosis partially via regulating the expression of ASK1. Ad-miR-19a significantly decreased the activity of a luciferase reporter containing the 3’UTR of ASK1. These results further suggest that, in LPS-treated endothelial cells, miR-19a controls ASK1 expression by regulating mRNA translation. Therefore, our study suggests a novel mechanism for the regulation of ASK1 expression at the translational level in response to inflammatory stimuli.

## Conclusions

In summary, our data suggest that miR-19a is expressed in HUVECs, and the expression of miR-19a is modulated by LPS. Moreover, we found that overexpression of miR-19a inhibits LPS-induced apoptosis of endothelial cells. Furthermore, we identified ASK1 as a direct target of miR-19a in HUVECs. MiR-19a regulates ASK1 expression by targeting specific binding sites in the 3’UTR of ASK1. Taken together, these results suggest that miR-19a may be a useful target to protect endothelial cells from LPS-induced apoptosis.

## Additional file

Additional file 1: Figure S1. MicroRNAs target to human ASK13’-UTR. Human ASK1 3’-UTR was analyzed using a website tools (http://www.microrna.org/microrna/getGeneForm.do). **Figure S2.** MiR19a inhibitor has no effect on expression of miR-20a. HUVECs were cultured in 6-well plate, after 80% confluence, cells were treated with miR-19a inhibitor or control inhibitor for 24 h. The expression of miR-20a was determined by quantitative real time PCR. **Figure S3.** MiR-19a suppressed ASK1 3’UTR-Luc activity. The ASK 3’UTR was cloned into pGL3-promoter luciferase reporter vector between luciferase coding sequence and SV40-poly(A) sequence using the XbaI site. EAhy926 cells transfected with pGL3 reporter vector fused with either ASK1 wild-type 3’UTR or ASK1 3’UTR mutant for 12 h, and then the cells were infected with Ad-LacZ or Ad-miR19a (50MOI). After 36 h cells were harvested and luciferase assay was performed. Luc-ASK1 3’UTR-M1: site-1 “UUGCAC” was mutated; Luc-ASK1 3’UTR-M2: site-2 “UUGCAC” was mutated; Luc-ASK1 3’UTR-MU, both sites were mutated. (* indicates P < 0.01 compared with Ad-LacZ group). **Figure S4.** LPS down-regulates expression of miR-19a and miR-20a in endothelial cells. HUVECs were cultured in 6-well plate, after 80% confluence, cells were treated with 100 ng/mL LPS for different times point as indicated. Quantitative real-time PCR showed that miR-19a and miR-20a expression were down-regulated. (* indicates P < 0.05 compared with miR-19a/LPS 0 h; # indicates P < 0.05 compared with miR-20a/LPS 0 h; § indicates P < 0.05 compared with miR-19a).
