# Supplementary material for: MicroRNA-19a regulates lipopolysaccharide-induced endothelial cell apoptosis through modulation of apoptosis signal-regulating kinase 1 expression
Source: BMC Mol Biol. 2015 May 16;16:11. doi: 10.1186/s12867-015-0034-8 (PMC4446110; doi:10.1186/s12867-015-0034-8)
Supplement: Additional file 1: Figure S1. — MicroRNAs target to human ASK13’-UTR. Human ASK1 3’-UTR was analyzed using a website tools (http://www.microrna.org/microrna/getGeneForm.do). Figure S2. MiR19a inhibitor has no effect on expression of miR-20a. HUVECs were cultured in 6-well plate, after 80% confluence, cells were treated with miR-19a inhibitor or control inhibitor for 24 h. The expression of miR-20a was determined by quantitative real time PCR. Figure S3. MiR-19a suppressed ASK1 3’UTR-Luc activity. The ASK 3’UTR was cloned into pGL3-promoter luciferase reporter vector between luciferase coding sequence and SV40-poly(A) sequence using the XbaI site. EAhy926 cells transfected with pGL3 reporter vector fused with either ASK1 wild-type 3’UTR or ASK1 3’UTR mutant for 12 h, and then the cells were infected with Ad-LacZ or Ad-miR19a (50MOI). After 36 h cells were harvested and luciferase assay was performed. Luc-ASK1 3’UTR-M1: site-1 “UUGCAC” was mutated; Luc-ASK1 3’UTR-M2: site-2 “UUGCAC” was mutated; Luc-ASK1 3’UTR-MU, both sites were mutated. (* indicates P < 0.01 compared with Ad-LacZ group). Figure S4. LPS down-regulates expression of miR-19a and miR-20a in endothelial cells. HUVECs were cultured in 6-well plate, after 80% confluence, cells were treated with 100 ng/mL LPS for different times point as indicated. Quantitative real-time PCR showed that miR-19a and miR-20a expression were down-regulated. (* indicates P < 0.05 compared with miR-19a/LPS 0 h; # indicates P < 0.05 compared with miR-20a/LPS 0 h; § indicates P < 0.05 compared with miR-19a). [file 12867_2015_34_MOESM1_ESM.docx]

**Supplement Figure and Figure Legends:**


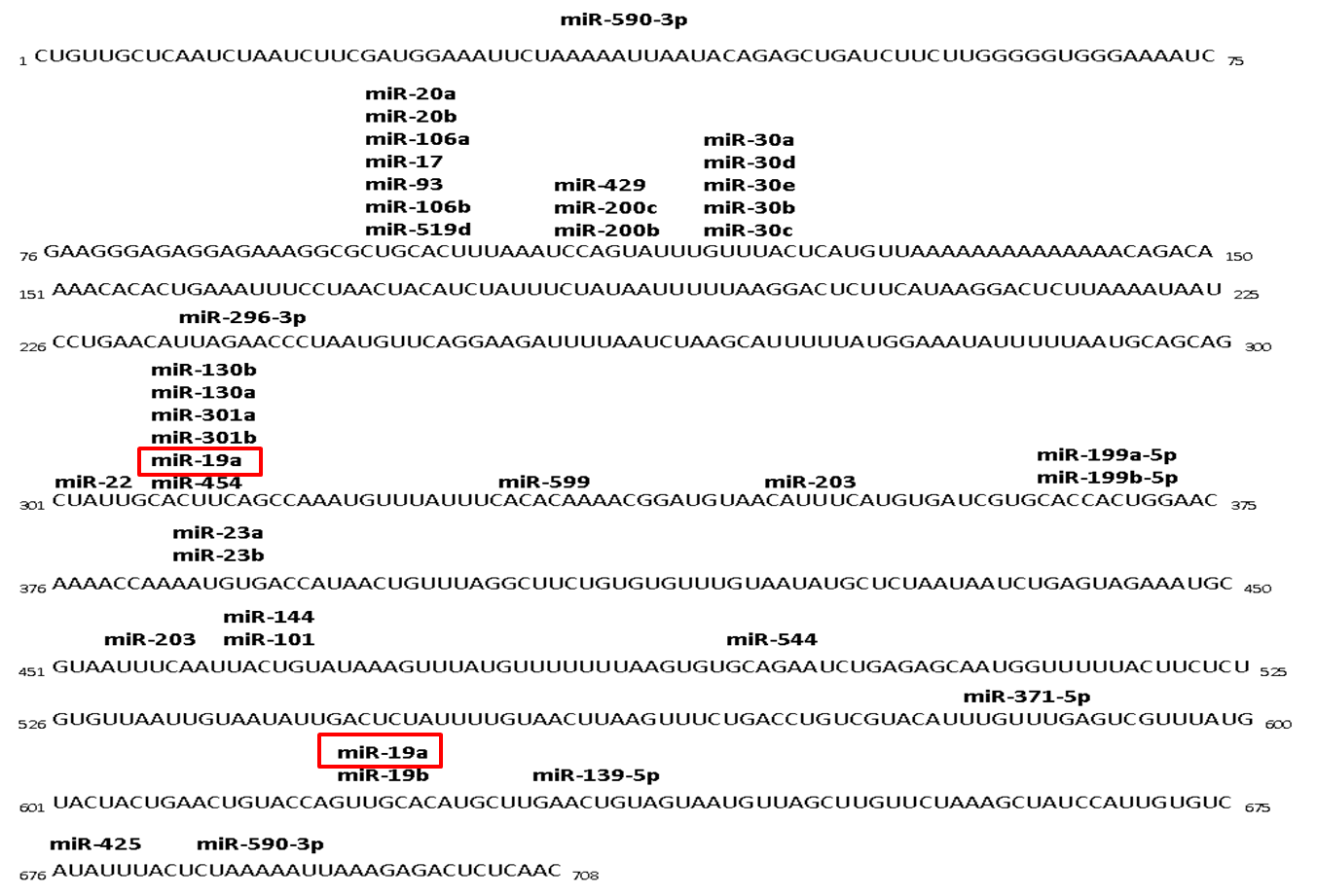


**Figure S1. MicroRNAs target to human ASK13’-UTR.** Human ASK1 3’-UTR was analyzed using a website tools (<http://www.microrna.org/microrna/getGeneForm.do>).


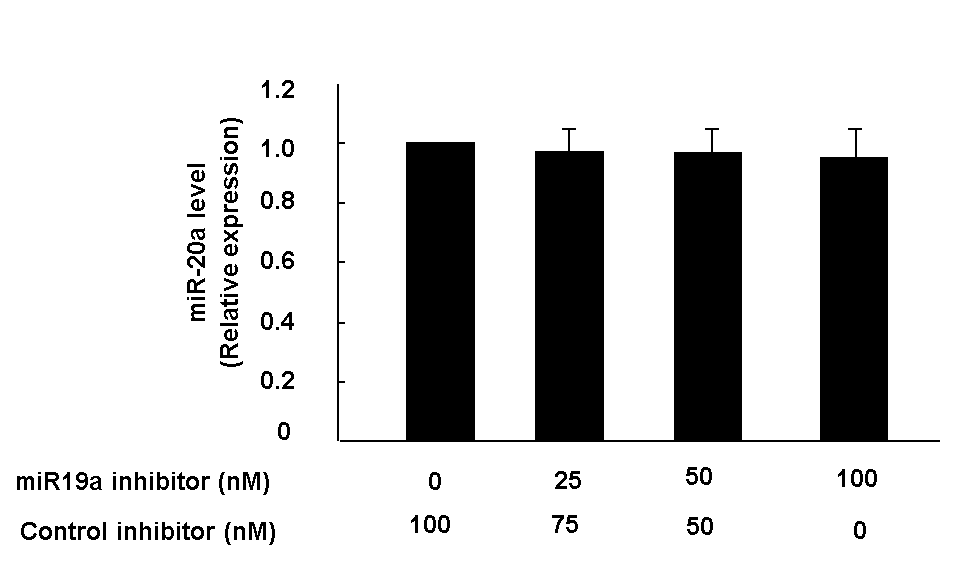


**Figure S2. MiR19a inhibitor has no effect on expression of miR-20a**. HUVECs were cultured in 6-well plate, after 80% confluence, cells were treated with miR-19a inhibitor or control inhibitor for 24h. The expression of miR-20a was determined by quantitative real time PCR.


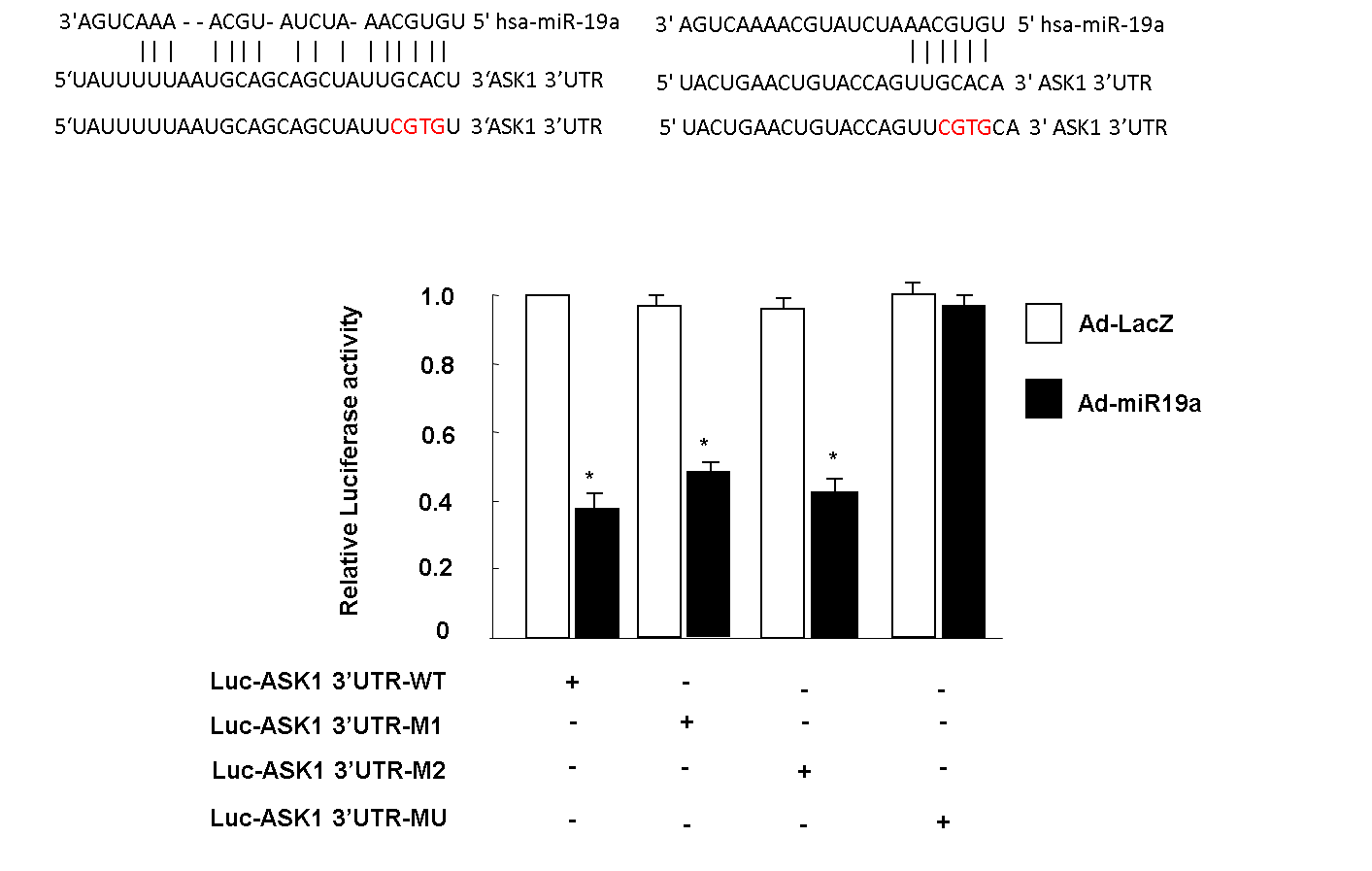


**Figure S3. MiR-19a suppressed ASK1 3’UTR-Luc activity.** The ASK 3’UTR was cloned into pGL3-promoter luciferase reporter vector between luciferase coding sequence and SV40-poly(A) sequence using the XbaI site. EAhy926 cells transfected with pGL3 reporter vector fused with either ASK1 wild-type 3’UTR or ASK1 3’UTR mutant for 12h, and then the cells were infected with Ad-LacZ or Ad-miR19a (50MOI). After 36h cells were harvested and luciferase assay was performed. Luc-ASK1 3’UTR-M1: site-1 “UUGCAC” was mutated; Luc-ASK1 3’UTR-M2: site-2 “UUGCAC” was mutated; Luc-ASK1 3’UTR-MU, both sites were mutated. ( * indicates *P<0.01* compared with Ad-LacZ group)


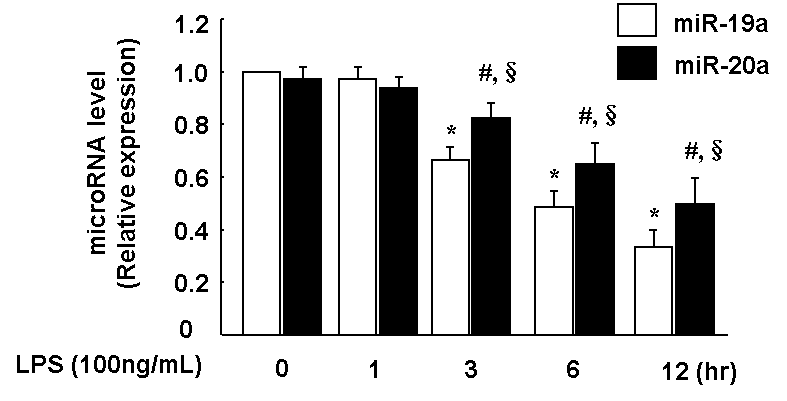


**Figure S4. LPS down-regulates expression of miR-19a and miR-20a in endothelial cells.** HUVECs were cultured in 6-well plate, after 80% confluence, cells were treated with 100ng/mL LPS for different times point as indicated. Quantitative real-time PCR showed that miR-19a and miR-20a expression were down-regulated.( * indicates *P<0.05* compared with miR-19a/LPS 0h; # indicates *P<0.05* compared with miR-20a/LPS 0h; § indicates *P<0.05* compared with miR-19a)
